# Influence of Epicuticular Physicochemical Properties on Porcine Rotavirus Adsorption to 24 Leafy Green Vegetables and Tomatoes

**DOI:** 10.1371/journal.pone.0132841

**Published:** 2015-07-16

**Authors:** Lu Lu, Kang-Mo Ku, Sindy Paola Palma-Salgado, Andrew Page Storm, Hao Feng, John A. Juvik, Thanh H. Nguyen

**Affiliations:** 1 Department of Civil and Environmental Engineering, University of Illinois at Urbana-Champaign, Urbana, IL, United States of America; 2 Department of Crop Science, University of Illinois at Urbana-Champaign, Urbana, IL, United States of America; 3 Department of Food Science and Human Nutrition, University of Illinois at Urbana-Champaign, Urbana, IL, United States of America; 4 Department of Molecular and Cellular Biology, University of Illinois at Urbana-Champaign, Urbana, IL, United States of America; The Australian National University, AUSTRALIA

## Abstract

Foodborne diseases are a persistent problem in the United States and worldwide. Fresh produce, especially those used as raw foods like salad vegetables, can be contaminated, causing illness. In this study, we determined the number of rotaviruses adsorbed on produce surfaces using group A porcine rotaviruses and 24 cultivars of leafy vegetables and tomato fruits. We also characterized the physicochemical properties of each produce’s outermost surface layer, known as the epicuticle. The number of rotaviruses found on produce surfaces varied among cultivars. Three-dimensional crystalline wax structures on the epicuticular surfaces were found to significantly contribute to the inhibition of viral adsorption to the produce surfaces (p = 0.01). We found significant negative correlations between the number of rotaviruses adsorbed on the epicuticular surfaces and the concentrations of alkanes, fatty acids, and total waxes on the epicuticular surfaces. Partial least square model fitting results suggest that alkanes, ketones, fatty acids, alcohols, contact angle and surface roughness together can explain 60% of the variation in viral adsorption. The results suggest that various fresh produce surface properties need to be collectively considered for efficient sanitation treatments. Up to 10.8% of the originally applied rotaviruses were found on the produce surfaces after three washing treatments, suggesting a potential public health concern regarding rotavirus contamination.

## Introduction

As a persistent problem in the United States, every year, foodborne pathogens cause illness for approximately 48 million Americans, and among these cases, 3,000 are fatal [1]. According to the U.S. Food and Drug Administration [2], foodborne illnesses are estimated to cost the U.S. economy $10–83 billion each year, from medical expenses, reduced productivity, and other costs. Of all foodborne illnesses in the U.S., human enteric viruses are a leading cause [3]. In a number of foodborne outbreaks, epidemiological evidence suggests that contaminated produce surfaces during pre- and post-harvest are critical in viral transmission [4]. Produce contamination before harvest, such as being irrigated or spayed with contaminated water, is an important safety concern, especially for raw and fresh produce [5–10]. Although certain inactivation strategies such as thermal inactivation and high hydrostatic pressure are effective in viral inactivation, they are mostly not applicable to fresh vegetable produce used for raw consumption because they either cook the produce or cause significant tissue damage and changes in produce appearance and taste [11, 12]. In the U.S., reports of foodborne illnesses associated with contaminated raw produce have increased, with salad greens and fruits found as the vehicles for pathogen transmission [13]. Despite the role of fresh produce in viral transmission, only limited information is available concerning the factors controlling viral adsorption to produce surfaces.

Several factors, including surface charge, roughness, and hydrophobicity, were found to contribute to viral adsorption to produce [14–17]. Ionic strength and pH of solutions containing viruses and temperature also influenced viral adsorption and survival [15, 17, 18]. The presence of stomata and exposed carbohydrates of plant cell walls were found to enhance adsorption of viruses to romaine lettuce [19–21]. Produce surfaces or cuticles are composed of various layers that serve separate functions in plant development, protection, and adaptation to the growing environment [22]. The outermost layer of produce surfaces is composed of epicuticular waxes and is the most likely to interact with microorganisms attaching to produce surfaces. The epicuticular waxes were proposed to act as a physical barrier to prevent infection by plant viruses or fungi, in addition to other critical functions, including drought tolerance and tissue protection from ultraviolet light [23–25]. While previous studies show an important role of the physicochemical characteristics of produce surfaces for several species influencing viral adsorption, a comprehensive study of the major leafy green salad species commonly consumed by humans as fresh or minimally processed produce has not been conducted.

To fill this knowledge gap, we conducted a study to determine the correlations between viral adsorption and physicochemical characteristics of produce surfaces using 24 cultivars of leafy green vegetables and tomato fruits. Group A rotavirus, the most common cause of severe diarrhea in children under the age of five and of gastroenteritis among all ages [4, 10, 26], was selected as a model enteric viral pathogen. Although a rotavirus vaccine was developed and applied in both developed and developing countries, in such areas as Africa and Asia, rotavirus is still a major cause of pediatric gastroenteritis. Rotavirus vaccine efficacy is only 48% in Asia and 30% in Africa, with the lowest efficacy found in Mali (17%) [27–30]. Group A rotaviruses were detected in wastewater and surface water in Kenya [31], irrigation water and even receiving vegetables in South Africa [10]. Group A porcine rotavirus (OSU strain), a surrogate for human rotavirus Wa, was used in our study due to its stability and similar structural proteins to the human rotavirus strain Wa [32]. Twenty-four different cultivars of leafy greens and tomatoes were grown in the greenhouse and used as model produce. Compared to previous work [14–20, 33], the greater number of cultivars from various species in this study provides more comprehensive information on viral adsorption to produce and generates a larger database for future investigations. Undamaged vegetable leaves and tomato fruit skins were characterized and tested in our viral assays to generate correlations between viral adsorption and physicochemical characteristics of the outermost layers of produce surfaces. The produce surface hydrophobicity, roughness, stoma number and length, the presence or absence of 3-D epicuticular wax crystals, and the chemical compositions of the epicuticular wax layers were determined based on the measurements of contact angle, laser confocal microscopy, scanning electron microscopy (SEM), and chemical identification using gas chromatography.

## Materials and Methods

### Greenhouse production of leafy vegetables and tomato fruits

Twenty-one leafy green cultivars and three tomato cultivars with expected substantially different physical and chemical characteristics were selected to provide the statistical rigor to correlate the cuticular chemical and topographical variables (wax chemistry and concentrations, hydrophobicity, roughness, and solute contact angle) with viral adsorption. Greenhouse conditions were consistently maintained throughout the study so that produce replicates over many months can be obtained. The vegetable cultivars are listed in S1 Table. All seeds were purchased from Johnny’s Selected Seeds (Winslow, ME). Seeds of each cultivar were germinated in 32-cell plant plug trays filled with sunshine LC1 (Sun Gro Horticulture, Vancouver, British Columbia, Canada) professional soil mix. Seedlings were grown in a greenhouse at the University of Illinois at Urbana-Champaign under a 25°C/17°C and 14 h/10 h: day/night temperature regimen with supplemental lighting. Twenty days after germination, seedlings were transferred to 4 L pots. Information from the seed company was used to determine appropriate harvest dates for the leafy vegetables and tomato fruits (S1 Table). Leaves of Tokyo bekana, ‘Rhodos’ endive, ‘Southern Giant Curled’ mustard, Mizuna, ‘Tyee’ spinach, ‘Racoon’ spinach, ‘Carmel’ spinach, Tatsoi, and Arugula were harvested 40–45 days after sowing seeds. Leaf tissues of ‘Top Bunch’ collards, ‘Starbor’ kale, ‘Red Russian’ kale, ‘Totem’ Belgian endive, ‘Two Star’ lettuce, ‘Tropicana’ lettuce, and ‘Outredgeous’ romaine lettuce were harvested 50–65 days after sowing seeds. Leaves from median internodes from each leafy vegetable were harvested for analysis. Heads of ‘Perseo’ radicchio, four cabbage cultivars, and fruits of the three tomato cultivars, shown in S1 Table, were harvested 66–85 days after sowing seeds. The greenhouse is disinfected regularly allowing us to grow vegetables without substantial contamination by bacteria and other microorganisms that may change the health of vegetables. The produce was harvested at market maturity.

### Contact angle

At the harvest time, a total of six leaves or fruits (two from each of three plants) were collected and immediately transported to a processing facility where they were stored at 5 ± 1°C prior to contact angle measurement. A 15.6 mm diameter sterile cork borer was used to excise two disks from each leaf or fruit. These disks were then taped (3M, Minnesota, USA) to a smooth microscope glass slide exposing either the abaxial or adaxial surface of leaves (tomato fruits had only one surface, adaxial). Glass slides were covered with a moistened paper towel and clear plastic wrap to prevent dehydration of leaf or fruit disks. The water contact angle was obtained using a goniometer (KSV Instruments, Stockholm, Sweden) model CAM 200. Using a pipette, 5 μl of deionized water was placed on the center of each leaf disk and within 20 seconds five contact angle readings were measured.

### Surface roughness and stoma number

Produce samples were prepared following the same procedure used for contact angle measurement. A confocal microscope (NanoFocus, μSurf explorer) was used to determine 3-dimensional surface parameters. Area-average root mean square roughness ($\overset{-}{S}$_q_ bar) was obtained from the average of a number of linear root mean square roughness S_q_ measured from the 3-D image reconstructed from 2-D laser confocal images over an area of 0.3 mm × 0.3 mm (0.09 mm^2^). S_q_ is the geometric average height of roughness-component irregularities from the mean line measured along one line on the 3-D image. Image analysis was done using the software Mountains (Digital, Surf). The number of stomata was also counted from the same pictures taken by a NanoFocus confocal microscope.

### Image of epicuticular surface and stoma length determination by scanning electron microscopy

Fresh produce samples were freeze-dried and sputter-coated using a previously described protocol [34]. The vegetable leaf and fruit samples were sputter-coated with AU/PU metal ions (K575 sputter, Emitech Ltd, Ashford, Kent, UK). Images of the epicuticular surface were taken using a JSM-6060LV scanning electron microscope (SEM; JEOL Ltd, Tokyo, Japan). The images were captured at 500 × resolution from at least three different leaves or fruits. Some sample images of the epicuticular wax crystals were taken at 2,000 × resolution or higher. Stoma lengths were measured using ImageJ software version 1.48. To confirm the epicuticular wax extraction efficiency described below, before and after extraction, images of leaf surfaces from collards and Starbor kale were also taken by SEM.

### Epicuticular chemical composition

Healthy and undamaged vegetable leaves and tomato fruits at market maturity were harvested from each plant cultivar. Two leaf disks were cut from the leaves of three separate plants (biological replicate samples) using a cork borer (diameter 17 mm). For extraction of epicuticular waxes, two leaf disks were put into a 20 ml glass vial (Fisher Scientific, Waltham, MA) and 5 ml of chloroform was added for extraction from both sides of the disks for 1 min at room temperature. One hundred μl of *n*-tetracosane (1 mg/ml) was added to the 5 ml of chloroform solvent as an internal standard. For epicuticular wax extraction of tomato fruits, entire tomato fruits were added into either 200 mL beakers or 50 ml test tubes filled with chloroform solvent containing *n*-tetracosane (1 mg/ml) internal standard. 0.3 ml of wax extract from each produce sample was transferred to Reacti-vial (Thermo Fisher Scientific Inc., Waltham, MA) and subsequently evaporated under a gentle stream of nitrogen and re-dissolved in a mixture of 50 μl of pyridine and 100 μl of bis-*N*, *N*- (trimethylsilyl) trifluoroacetamide containing 10% trimethylchlorosilane (TMCS; Sigma-Aldrich, St. Louis, MO). The wax solutions were heated at 75°C for 75 min to convert waxes into volatile trimethylsilyl derivatives. One μl of the solutions was injected into a Hewlett Packard HP 5890 Series-I GC system equipped with a single flame-ionization detector (FID; Agilent Technologies, Santa Clara, CA) and 30 m × 0.25 mm SLB-5ms capillary column (Supelco Inc, Bellefonte, PA) to determine the wax concentration and composition. Qualitative and quantitative composition analyses were conducted as described previously based on *n*-tetracosane as an internal standard [35]. Relative response factors of *n*-tetracosane to each standard wax compound were calculated using ChemStation software B02.01 (Agilent Technologies) for the wax quantification. Nonacosan-15-one was identified using a 6890N GC coupled to an HP-5973N MS detector, according to a previously published study [36]. Since nonacosan-15-one standard was not commercially available, heptacosane-14-one was used as a standard, and concentrations of nonacosan-15-one were quantified based on the effective carbon number concept [37].

### MA-104 cell culture and OSU rotavirus propagation and purification

Group A porcine rotaviruses OSU strain (ATCC # VR-892) were propagated by infecting embryonic African green monkey kidney cells (MA-104 cells) grown in minimum essential medium (MEM, Gibco) containing 5% fetal bovine serum (FBS) [38, 39]. Porcine rotaviruses OSU strain and MA-104 cells were purchased from ATCC (Manassas, VA). Before virus infection, the MA-104 cells were grown in roller bottles with an inner surface area of 850 cm^2^ (Thermo Fisher Scientific Inc., Waltham, MA) and incubated at 37°C under 5% CO_2_ for 5–6 days. The medium was replaced by fresh medium on the third day of incubation. After a confluent cell monolayer was visible on the bottle wall, the medium was removed, and the cells were washed twice with phosphate-buffered saline (PBS) solution. After the PBS buffer was removed, rotaviruses were activated with 10 μg/ml of trypsin for 30 min. The viruses were added to maintain around 2–5 focus-forming units per cell (FFU/cell). After 90 min of incubation at 37°C under 5% CO_2_, the cells were washed twice with PBS and then incubated in MEM without the serum for 16 to 18 h. Once the infected cells were fully detached from the bottle wall, the cells and viral solution were collected and stored at 4°C until further purification. The rotavirus solution underwent three sequential freezing and thawing treatments at -80°C and 37°C. Then, the viruses were separated from the cell debris by centrifugation at 1000 × g for 10 min at 20e cell debris by centrifugation at 1000 e wall, incubation. After a confluent cell monolayer was visiblThermo Scientific, Nalgene, Rochester, NY) to further remove cell debris. Then, the filtrate was subjected to membrane dialysis using a 100 kDa ultrafiltration membrane (Koch Membrane, polymer polyvinylidene fluoride; Koch, Wilmington, MA) in an Amicon stirred cell (Millipore) to remove the medium and to concentrate the viruses, as described in previous work [40]. In this dialysis membrane system, the rotaviruses were retained on the membrane surface and subsequently washed with a solution containing 0.1 mM of CaCl_2_ and 1 mM NaHCO_3_. This concentration of Ca^2+^ was used to keep the rotavirus capsid stable [41].

The infectivity assay (FFU assay) was adopted from previous publications [38, 39], and the protocol is briefly described here. The rotavirus stock was treated with 10 μg/ml of trypsin for 30 min and made into a series of dilutions in serum-free MEM. After confluent MA104 monolayers in a 96-well plate had been rinsed twice with PBS, 50 μl of each diluted virus solution from the rotavirus stock was applied to the monolayers and incubated at 37°C under 5% CO_2_ for 30 min. Afterward, the virus solutions were removed from the plate and the cell monolayers were washed twice with serum-free MEM. The cells were then incubated with 100 μl of serum-free MEM in each well at 37°C under 5% CO_2_ for 18 h prior to immunocytochemical quantification of infected cells by rotaviruses.

After 18-h incubation, the cell monolayers were rinsed twice with PBS, and fixed with 9:1 methanol (Sigma-Aldrich, St. Louis, MO)–glacial acidic acid (Fisher Scientific, Waltham, MA) for 2 min. The monolayers were then hydrated with 70% and 50% ethanol subsequently for 5min, and then subjected for 10-min treatment with 3% H_2_O_2_ (30%; Fisher Scientific, Waltham, MA) diluted in wash buffer (125 mM Tris (Fisher Scientific, Waltham, MA), 350 mM NaCl (Fisher Scientific, Waltham, MA), and 0.25% Triton X-100 (Sigma-Aldrich, St. Louis, MO); pH = 7.6). Afterward, the cells were rinsed with wash buffer for 10 min and incubated with 5% normal goat serum (Vector Laboratories, Burlingame, CA) for 20 min to block nonspecific bindings of primary antibodies. After this step, the primary antibodies (Dako, Carpinteria, CA; catalog no. B218) diluted 1:100 in wash buffer were applied to the monolayers and incubated at 37°C for 1 h. After being rinsed twice with wash buffer, the cells were incubated with the secondary antibodies (biotinylated goat anti-rabbit immuno-globulin G; Vector Laboratories, Burlingame, CA) diluted 1:200 in wash buffer for 20 min. Two washings with wash buffer were applied to the monolayers after this step. After washing, the ABC reagent (Vector Laboratories, Burlingame, CA), made 30 min prior to use and diluted as 1 (reagent A):1 (reagent B):50 (wash buffer), was applied to the monolayers for 20 min. Afterward, the cells were rinsed twice with wash buffer and then incubated with the stain peroxidase chromogen (KPL, Gaithersburg, Maryland) for less than 9 min to avoid nonspecific cell staining. Deionized (DI) water was then added to each well, and the brown-stained cells, which were the infected cells by rotaviruses, were quantified using an inverted microscope. This assay using a 96 well plate has a detection limit of 1200 FFU (50 μl of 2.4 ×10^4^ FFU ml^-1^ viral solution).

### OSU rotavirus adsorption assay to leafy vegetables and tomato fruits

**Fig 1:**
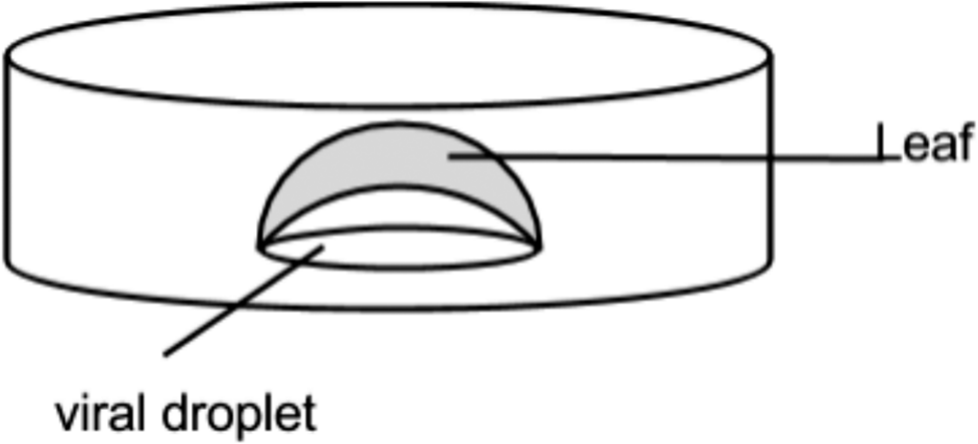
Experimental schema for the OSU rotavirus adsorption assay to leafy vegetables and tomato fruits. Each piece of vegetable leaf or tomato fruit skin was transferred carefully and gently onto the top of a droplet of 300 μl OSU rotavirus solution in PBS on a 35-mm-glass-bottom-dish. The edge effects (viruses might diffuse into the piece through its edge) were avoided by cutting the piece into a smaller disk for later RNA extraction.

Harvested vegetable heads, leaves, or tomato fruits were rinsed with DI water to remove soil particles attached to their surfaces and dried by gently laying a Kimwipe (Kimberly-Clark, Irving, TX) on the adaxial surface. When no water was visible on the produce surfaces, two 15.6 mm diameter disks were excised from each leaf and fruit sample. For each cultivar, two leaves, heads, or fruits from three different plants were harvested for 6 replicate measurements. Each piece was gently transferred, with a tweezer, onto the top of a droplet of 300 μl diluted porcine rotavirus solution in PBS on a 35-mm-glass-bottom-dish (MatTek Corporation, Ashland, MA). See Fig 1 for the experimental schema. Rotavirus concentration in this droplet was determined to be 7.17 ± 0.05 log_10_ genome copies/ml (N = 4) by RT-qPCR (see below). The petri dish containing the produce piece and the viral droplet was loosely capped and incubated for 2 h at room temperature in a biological cabinet. After this incubation period, the produce pieces were transferred with a tweezer, in the same way as described above, to a 24-well plate, which had 700 μl PBS in each well. The plate was gently shaken for 15 s, and the PBS solution was then carefully removed. This washing step was repeated three times before the produce pieces were removed from the well plate and the disks were excised with another corker borer (with a diameter of 11.1 mm) into smaller diameter pieces to remove the cut edges. Since viruses might be attached to the edges during the incubation period or washing steps, this treatment was important to avoid potentially confounding results. Each piece was transferred with a tweezer into a 1.7 ml labeled tube for RNA extraction and RT-qPCR. The adaxial surfaces were kept facing up throughout the whole experiment, except during incubation and washing.

The negative controls for these assays underwent the same procedure except that they were incubated on PBS droplets without porcine rotaviruses for 2 h. Based on the results obtained from the negative controls, we concluded that the leaves were not previously contaminated with rotaviruses. Due to the pool of 24 cultivars whose mature tissues were available at different times, the viral assays were conducted over an 8-week period from March to May, 2014. All viral adsorption experiments were conducted using the same OSU rotavirus stock (see below for concentration determination). Infectivity assays showed that the OSU rotavirus stock had approximately 5×10^7^ FFU/ml. Infectivity assays were also conducted for the viral adsorption experiments, however, the concentration of infective rotaviruses on the produce samples was generally below the detection limit of the infectivity assay using a 96 well plate (2.4 ×10^4^ FFU ml^-1^ viral solution). While the infectivity assay may be sensitive to aggregation of viruses, the qPCR method is not because it is based on the extracted genomes of all viruses.

### OSU viral RNA extraction from inoculated produce surfaces

The RNA extraction was conducted with E.Z.N.A Total RNA Kit I (Omega, Norcross, GA) following the manufacturer’s protocol in a sterilized RNA extraction cabinet to avoid RNA contamination and degradation. The extracted RNA was dissolved in diethylpyrocarbonate (DEPC) water and stored at -80°C before quantification by real-time quantitative PCR (RT-qPCR).

### Detection of OSU rotavirus adsorption by RT-qPCR

We first determined the concentration of the OSU rotavirus stock (8 log_10_ genome copies/ml) by conducting one-step RT-qPCR in parallel with a standard calibration curve for known concentrations of a plasmid cDNA standard (2207 bp) containing the inserted rotavirus NSP3 gene (212 bp). The ‘JVK’ Primers (Forward: CAGTGGTTGATGCTCAAGAT and Reverse: TCATTGTAATCATATTGAATACCCA) were used as described in previous studies [42, 43] to specifically amplify the NSP3 gene of the OSU rotaviruses. The primers and the plasmid cDNA standards were purchased from Integrated DNA Technologies (Coralville, IA). The concentration of dissolved plasmid DNA in DI water was measured by Qubit dsDNA HS assay kit (Life Technologies, Grand Island, NY), according to the manufacturer’s manual. The measured cDNA standard concentration (1.88 μg/ml) was then converted into copy numbers (11.9 log_10_ genome copies/ml). For experiments with plant tissues, the extracted RNA from the OSU stock with known concentration (8 log_10_ genome copies/ml) was used to determine a detection limit of 3.9 log_10_ genome copies/ml with the corresponding Ct value at 34.3 ± 0.1 (N = 3).

The number of adsorbed rotaviruses on each produce sample surface was determined by one-step RT-qPCR using an iTaq One-Step Universal SYBER RT-qPCR kit (Bio-Rad Laboratories, Hercules, CA). The overall volume of each qPCR reaction was 10 μl, composed of 2 × iTaq Mix, 0.3 mM of each primer, 125 × iScript reverse transcriptase, 3 μl RNA template, and DNase/RNase-free distilled water. The prepared PCR reactions were conducted with a Bio-Rad Unicon qPCR machine (Hercules, CA). The qPCR program was 48°C, 10 min (reverse transcription), and 95d 0 min (reverse transcription), PCR program was 48was 48TechnologiiTaq polymerase), with cycles of 95°C, 15 s (melting DNA double strands), 54°C, 20 s (primers annealing), 60°C, 30 s (elongation), 60–95°C, and 0.05 s increments. The PCR specificity was checked on a gel after qPCR, and only one band at around 200bp was observed under a Bio-Rad Universal Hood II Imager (Hercules, CA). The number of RNA genome copies from OSU rotaviruses adsorbed to each sample disk was calculated via equations obtained from standard curves conducted for every set of qPCR. For example, Y = −3.497X + 47.536 (R^2^ = 0.99, efficiency = 93%). Y is the RNA amount (log_10_ genome copies/ml), and X is the Ct value. The number of adsorbed OSU rotaviruses was expressed as log_10_ genome copies normalized by the produce sample area in cm^2^.

Leaves from two cultivars with epicuticular wax crystals (‘Top Bunch’ collards and ‘Red Russian’ kale) and two cultivars without epicuticular wax crystals (‘Two Star’ lettuce, and ‘Totem’ Belgian endive) were selected as additional controls for RT-qPCR inhibitors. RT-qPCR inhibitors were tested by spiking RNA extracted from the OSU virus stock into the extracted solutions from these four cultivars used as controls. The measured Ct values from these four controls were compared with those determined for the extracted RNA at the same concentration. The extracted RNA with a concentration of 5.3 log_10_ genome copies /mL showed an average Ct value of 28.72 ± 0.4 (N = 6), while the controls with the same concentration had Ct values of 28.89 ± 0.2 (N = 8). No significant difference was found between these two sets of Ct values (P = 0.35), and therefore no inhibitors were present in this system. The negative controls for rotavirus adsorption assays showed their Ct values as “NA”, indicating no rotaviruses present on the 24 vegetables prior to the viral adsorption assays. The same Ct readings (“NA”) were obtained for qPCR negative controls, which used DNase/RNase-free distilled water as templates, suggesting no contamination in the qPCR reactions.

### Statistical analysis

Statistical analyses were conducted using the JMP 10 software package (SAS institute Inc., Cary, NC). Data were subjected to analysis of variance (one-way ANOVA model). A primary source of variation came from using different vegetable cultivars from various species. The means were compared by the least significant difference (LSD) test at a significance level of *P* = 0.05. A threshold of ± 0.404 and ± 0.433 was set for significant correlations with either 24 cultivars or 21 cultivars (tomatoes not included), respectively. Pearson’s correlation analysis was conducted among different variables. A partial least squares (PLS) model was established to predict the number of adsorbed OSU rotaviruses expressed as log_10_ genome copies normalized by the produce sample area in cm^2^ (Y variable) by specifying six variables (X variables: alkane, fatty acid, alcohol, and ketone concentrations, contact angle, and surface roughness) in a non-linear iterative partial least squares (NIPALS) algorithm after centering and scaling options in JMP. “Leave-One-Out” statistical approach was selected as cross validation in the PLS model. The cut-off value of 0.8 for variable importance for projection (VIP) was used to separate terms that do not make an important contribution to the dimensionality reduction involved in PLS [44].

## Results

### Contact angle and roughness for the studied plants

It is commonly accepted that a surface is hydrophobic when the static contact angle made between a water droplet and the surface is greater than 90° [45]. From the contact angles listed in Table 1, 12 leafy vegetables among the 24 studied cultivars were hydrophobic on both adaxial and abaxial leaf surfaces, whereas seven vegetables had hydrophilic surfaces on both sides of the leaf. The two highest contact angles on adaxial surfaces were observed on ‘Starbor’ kale (128.9° ± 9.8°) and ‘Red Russian’ kale (125.1° ± 4.9°) as shown in Table 1. Across all leafy cultivars, contact angle values between adaxial and abaxial leaf surfaces were not significantly different in a paired T-test (P = 0.13).

**Table 1:** Physical properties of epicuticular layers of 24 vegetable leaves and tomato fruits.

Sample name	Genus	Species	Contact angle-ad (˚)	Contact angle-ab (˚)	Surface roughness-ad (μm)	Surface roughness-ab (μm)	No. of stoma-ad^a^	No. of stoma-ab^a^	Stoma length (μm)
Tokyo bekana	*Brassica*	*rapa*	95.6 ± 7.1	95.6 ± 21.0	2.7 ± 0.9	7.4 ± 3.4	16.7 ± 5.5	24.7 ± 2.5	11.3 ± 1.0
‘Perseo’ radicchio	*Cichorium*	*intybus*	53.1 ± 15.0	55.6 ± 2.6	4.1 ± 2.0	2.9 ± 0.8	6.0 ± 3.0	14.3 ± 2.5	18.3 ± 0.6
‘Rhodos’ endive	*Cichorium*	*endivia*	52.7 ± 8.5	44.6 ± 3.5	5.4 ± 4.0	6.0 ± 2.7	1.0 ± 0.0	5.7 ± 1.2	12.9 ± 0.7
‘Southern Giant Curled’ mustard	*Brassica*	*juncea*	100.2 ± 4.6	116.9 ± 11.2	8.0 ± 0.6	8.9 ± 2.2	12.0 ± 3.6	21.3 ± 1.2	15.3 ±1.4
Mizuna	*Brassica*	*rapa*	93.1 ± 3.9	96.4 ± 0.7	2.5 ± 0.2	5.0 ± 0.9	26.7 ± 4.6	38.7 ± 8.1	7.8 ± 0.6
‘Tyee’ spinach	*Spinacia*	*oleracea*	99.3 ± 5.8	99.5 ± 13.9	3.5 ± 1.9	2.9 ± 0.2	13.7 ± 0.6	17.0 ± 4.0	11.3 ± 1.6
‘Racoon’ spinach	*Spinacia*	*oleracea*	104.2 ± 3.0	110.0 ± 2.4	2.8 ± 0.2	3.9 ± 0.8	8.0 ± 1.0	11.7 ± 1.2	10.7 ± 0.7
‘Carmel’ spinach	*Spinacia*	*oleracea*	87.7 ± 7.0	99.8 ± 2.7	3.0 ± 0.7	4.1 ± 1.4	10.0 ± 1.0	12.3 ± 3.2	10.3 ± 1.0
Tatsoi	*Brassica*	*rapa*	73.8 ± 16.2	85.2 ± 4.3	6.7 ± 0.9	3.1 ± 0.8	10.0 ± 4.4	20.0 ± 7.8	10.3 ± 1.3
‘Top Bunch’ collards	*Brassica*	*oleracea*	115.1 ± 4.5	127.6 ± 11.3	1.4 ± 0.5	1.9 ± 0.9	30.3 ± 1.5	32.7 ± 4.5	8.4 ± 0.8
‘Starbor’ kale	*Brassica*	*oleracea*	128.9 ± 9.8	126.3 ± 3.7	1.4 ± 0.1	1.6 ± 0.4	21.0 ± 5.2	20.0 ± 1.0	7.5 ± 0.6
‘Red Russian’ kale	*Brassica*	*napus*	125.1 ± 4.9	130.4 ± 7.5	2.1 ± 0.3	3.6 ± 1.3	11.7 ± 3.2	9.3 ± 0.6	13.2 ± 1.4
Arugula	*Eruca*	*sativa*	92.4 ± 8.3	96.3 ± 3.3	5.3 ± 1.2	7.4 ± 1.8	9.3 ± 1.5	18.3 ± 3.2	22.3 ± 1.5
‘Totem’ Belgian endive	*Cichorium*	*intybus*	56.5 ± 1.6	43.9 ± 2.9	4.0 ± 0.9	3.3 ± 0.9	8.0 ± 3.0	13.3 ± 1.2	12.8 ± 2.2
‘Two Star’ lettuce	*Lactuca*	*sativa*	49.1 ± 9.5	53.4 ± 2.5	2.6 ± 0.6	5.0 ± 1.6	4.0 ± 1.0	6.0 ± 1.7	14.2 ± 2.4
‘Tropicana’ lettuce	*Lactuca*	*sativa*	53.5 ± 10.3	67.4 ± 6.6	2.9 ± 1.2	7.1 ± 0.1	8.3 ± 2.1	8.7 ± 4.2	19.3 ± 1.3
‘Outredgeous’ romaine lettuce	*Lactuca*	*sativa*	60.2 ± 8.6	59.4 ± 6.5	2.5 ± 0.5	2.5 ± 0.8	13.7 ± 8.0	17.7 ± 4.6	14.5 ± 1.9
‘Super Red’ cabbage	*Brassica*	*oleracea*	77.9 ± 2.5	103.4 ± 4.6	1.3 ± 0.6	1.7 ± 0.9	16.0 ± 5.7	18.0 ± 5.7	11.7 ± 1.6
‘Gonzales’ cabbage	*Brassica*	*oleracea*	115.5 ± 1.9	107.3 ± 2.4	1.6 ± 0.2	1.9 ± 0.5	25.0 ± 7.1	18.0 ± 7.5	10.8 ± 1.2
‘Ruby Perfection’ cabbage	*Brassica*	*oleracea*	115.3 ± 3.0	119.1 ± 2.0	2.4 ± 0.3	1.4 ± 0.3	10.0 ± 1.7	14.7 ± 1.5	15.4 ± 0.9
‘Alcosa’ cabbage	*Brassica*	*oleracea*	110.8 ± 5.4	115.0 ± 5.1	6.0 ± 2.6	4.6 ± 0.9	21.7 ± 2.9	24.0 ± 0.0	8.3 ± 2.3
‘Sun Gold’ cherry tomatoes	*Solanum*	*lycopersicum*	85.4 ± 4.4	-	1.1 ± 0.5	-	-	-	-
‘Indigo Rose’ tomatoes	*Solanum*	*lycopersicum*	97.9 ± 10.6	-	7.1 ± 1.8	-	-	-	-
‘Rose’ tomatoes	*Solanum*	*lycopersicum*	110.7 ± 10.9	-	2.9 ± 0.4	-	-	-	-
LSD			14.9	13.6	2.2	2.9	8.5	7.4	2.3

(Contact angle is presented in °, and roughness is in μm.). Ad and ab indicate adaxial and abaxial leaf, respectively. Stoma lengths were measured on adaxial leaf surfaces.

Surface roughness was measured by laser beam scanning over an area of 300 μm × 300 μm and thus only reflected the vertical height variation of the produce surfaces within an area of 90,000 μm^2^. As shown in Table 1, among the 24 vegetables, the roughest surface was observed on mustard greens (8.0 μm ± 0.6 μm on the adaxial and 8.9 μm ± 2.2 μm on the abaxial surface) while cherry tomatoes had the smoothest surface (1.1 μm ± 0.5 μm). The three spinach cultivars showed similar roughness values within the group, as well as between the adaxial and abaxial surfaces of each cultivar. The lettuce group had similar adaxial surface roughness for all three members, whereas ‘Tropicana’ lettuce had rougher abaxial surfaces (7.1 μm ± 0.1 μm) than the other two lettuces (‘Two Star’ lettuce: 5.0 μm ± 1.6 μm; ‘Outredgeous’ romain lettuce: 2.5 μm ± 0.8 μm). ‘Alcosa’ cabbage displayed high roughness values on both adaxial and abaxial surfaces, while the other 3 cabbages had relatively smooth surfaces. Similarly, ‘Indigo Rose’ was the only tomato fruit cultivar with comparatively rough surfaces (7.1 μm ± 1.8 μm). Besides the roughness differences across the 24 vegetable cultivars, different roughness values were typically observed between adaxial and abaxial surfaces of the same genotype. ‘Tokyo’ bekana, ‘Tropicana’ lettuce, and Arugula had rougher abaxial surfaces than adaxial surfaces, while Tatsoi had rougher adaxial surfaces. Across all leafy cultivars, there was a significant correlation in surface roughness between adaxial and abaxial leaves (r = 0.584, P = 0.005). Across all cultivars, surface roughness values between adaxial and abaxial surfaces were significantly different based on results of a paired T-test (P = 0.009).

### Epicuticular surface chemical composition

The major epicuticular waxes on most tested plants in this study include long chain aliphatic alkanes, fatty acids, and alcohols. The concentration and composition of these waxes were determined from the surfaces of all 24 salad cultivars from various species (Table 2). ‘Top Bunch’ collard leaves (44.2 ± 4.8 μg/cm^2^), ‘Red Russian’ kale leaves (44.0 ± 2.2 μg/cm^2^), and ‘Alcosa’ cabbage heads (40.8 ± 4.3 μg/cm^2^) had significantly higher alkane concentrations than other cultivars, while ‘Two Star’ lettuce leaves (0.5 ± 0.3 μg/cm^2^), ‘Rhodos’ endive leaves (0.6 ± 0.6 μg/cm^2^), Mizuna leaves (0.9 ± 0.6 μg/cm^2^), and ‘Perseo’ radicchio leaves (1.0 ± 0.3 μg/cm^2^) had significantly lower alkane concentrations than other vegetables (Table 2). ‘Indigo Rose’ tomato fruits (18.4 ± 7.6 μg/cm^2^) had significantly higher fatty acid concentrations than other vegetables, whereas ‘Two Star’ lettuce leaves and ‘Rhodos’ endive leaves have even less than 1 μg/cm^2^ fatty acid concentrations. ‘Outredgeous’ romaine lettuce (15.3 ± 9.2 μg/cm^2^) leaves have the highest alcohol concentrations, while alcohols were not detected on the ‘Indigo Rose’ tomato fruits (Table 2). ‘Starbor’ kale leaves (35.5 ± 2.1 μg/cm^2^) had the highest ketone concentrations than other vegetables, followed by ‘Red Russian’ kale leaves (27.7 ± 0.9 μg/cm^2^), ‘Alcosa’ cabbage heads (26.2 ± 2.3 μg/cm^2^), and ‘Top Bunch’ collard leaves (24.1 ± 3.3 μg/cm^2^). ‘Rhodos’ endive leaves (3.5 ± 0.2 μg/cm^2^) and Mizuna leaves (4.4 ± 1.2 μg/cm^2^) had significantly lower total wax concentrations than other vegetables. ‘Red Russian’ kale (81.3 ± 3.7 μg/cm^2^), ‘Top Bunch’ collards (79.9 ± 9.2 μg/cm^2^), and ‘Starbor’ kale (78.4 ± 1.4 μg/cm^2^) leaves have the highest total wax content among the cultivars. Total epicuticular wax content ranged from 3.5 to 81.3 μg/cm^2^, as presented in Table 2.

**Table 2:** Chemical composition of epicuticular waxes from 24 vegetable leaves and tomato fruits and the genome copies from adsorbed rotaviruses on these produce surfaces.

	Epicuticular wax concentration (μg/cm^2^)					Viral copy numbers	
Sample name	Alkanes	Fatty acids	Alcohols	Ketones	Total wax content	Log _10_ copies/cm^2^	%^a^
Tokyo bekana	2.0 ± 0.6	1.6 ± 0.5	1.5 ± 0.3	0.2 ± 0.3	5.2 ± 2.7	4.5 ± 0.2	0.7 ± 0.3
‘Perseo’ radicchio	1.0 ± 0.3	1.8 ± 1.5	3.1 ± 0.4	0.0 ± 0.0	5.9 ± 3.9	4.9 ± 0.4	2.0 ± 1.1
‘Rhodos’ endive	0.6 ± 0.6	0.9 ± 0.3	2.1 ± 0.2	0.0 ± 0.0	3.5 ± 0.2	4.5 ± 0.2	0.8 ± 0.4
‘Southern Giant Curled’ mustard	3.8 ± 1.7	4.1 ± 1.9	1.6 ± 0.4	0.4 ± 0.3	9.8 ± 2.9	5.6 ± 0.1	9.3 ± 1.1
Mizuna	0.9 ± 0.6	2.5 ± 0.1	0.8 ± 0.3	0.0 ± 0.0	4.4 ± 1.2	5.2 ± 0.1	3.4 ± 0.9
‘Tyee’ spinach	5.8 ± 0.2	1.0 ± 0.1	2.9 ± 0.6	0.0 ± 0.0	9.7 ± 0.8	5.3 ± 0.2	4.2 ± 1.4
‘Racoon’ spinach	6.9 ± 0.4	1.2 ± 0.6	3.1 ± 0.3	0.0 ± 0.0	11.5 ± 0.5	5.6 ± 0.2	10.8 ± 7.4
‘Carmel’ spinach	6.1 ± 0.1	1.2 ± 0.4	2.8 ± 0.3	0.0 ± 0.0	10.0 ± 0.7	4.9 ± 0.1	1.6 ± 0.2
Tatsoi	2.7 ± 3.3	0.8 ± 0.2	2.0 ± 0.7	0.0 ± 0.0	5.7 ± 3.8	5.4 ± 0.1	6.1 ± 1.8
‘Top Bunch’ collard	44.2 ± 4.8	8.1 ± 1.1	3.3 ± 0.3	24.1 ± 3.3	79.9 ± 9.2	3.7 ± 0.1	0.1 ± 0.0
‘Starbor’ kale	35.0 ± 2.6	5.9 ± 4.3	1.9 ± 0.2	35.5 ± 2.1	78.4 ± 1.4	4.9 ± 0.2	2.1 ± 1.0
‘Red Russian’ kale	44.0 ± 2.2	6.3 ± 1.2	3.3 ± 0.1	27.7 ± 0.9	81.3 ± 3.7	4.7 ± 0.1	1.3 ± 0.4
Arugula	2.2 ± 0.9	6.4 ± 2.4	1.3 ± 0.5	0.5 ± 0.5	10.4 ± 3.2	5.0 ± 0.03	2.2 ± 0.2
‘Totem’ Belgian Endive	1.5 ± 0.2	1.7 ± 0.2	3.1 ± 0.2	0.0 ± 0.0	6.3 ± 0.2	5.2 ± 0.1	3.9 ± 1.2
‘Two Star’ lettuce	0.5 ± 0.3	0.7 ± 0.6	4.4 ± 1.2	0.0 ± 0.0	5.7 ± 2.0	4.6 ± 0.2	0.9 ± 0.4
‘Tropicana’ lettuce	1.3 ± 1.9	2.6 ± 2.1	6.1 ± 3.6	0.0 ± 0.0	10.1 ± 7.6	4.9 ± 0.1	1.7 ± 0.6
‘Outredgeous’ romaine lettuce	1.6 ± 0.8	2.9 ± 0.8	15.3 ± 9.2	0.0 ± 0.0	19.9 ± 8.2	4.1 ± 0.5	0.4 ± 0.4
‘Super Red’ cabbage	6.7 ± 2.4	2.2 ± 1.2	1.2 ± 0.6	2.6 ± 3.2	14.2 ± 5.6	4.9 ± 0.2	1.8 ± 0.8
‘Gonzales’ cabbage	15.7 ± 7.7	5.9 ± 3.3	0.6 ± 0.2	12.3 ± 6.4	34.5 ± 17.5	4.5 ± 0.1	0.8 ± 0.1
‘Ruby Perfection’ cabbage	5.3 ± 3.5	1.7 ± 1.1	0.9 ± 0.5	3.3 ± 2.3	11.5 ± 6.7	4.9 ± 0.3	2.1 ± 0.9
‘Alcosa’ cabbage	40.8 ± 4.3	6.6 ± 1.3	2.3 ± 0.4	26.2 ± 2.3	76.2 ± 7.5	4.4 ± 0.1	0.6 ± 0.2
‘Sun Gold’ cherry tomato	26.4 ± 0.9	5.6 ± 0.9	1.9 ± 0.6	0.0 ± 0.0	33.9 ± 1.0	3.9 ± 0.4	0.2 ± 0.2
‘Indigo Rose’ tomato	35.7 ± 7.6	18.4 ± 7.6	0.0 ± 0.0	0.0 ± 0.0	54.2 ± 2.0	4.2 ± 0.4	0.5 ± 0.6
‘Rose’ tomato	14.8 ± 0.9	6.9 ± 0.9	0.9 ± 3.3	0.7 ± 0.2	23.3 ± 0.1	4.4 ± 0.3	0.7 ± 0.5
LSD	4.86	4.02	3.47	2.98	9.33	0.261	15.1

^a^ The percentage was calculated using number of adsorbed rotaviruses divided by OSU rotavirus genome copies in the initial viral solution (7.17 ± 0.05 log_10_ genome copies/ml). Wax components were quantified by GC-FID and the total amount of wax was calculated as the sum of single components. LSD value was calculated by Student’s T-test at *P* = 0.05.

Of the six different genera tested in this study (*Brassica*, n = 11 cultivars; *Chchorium*, n = 3; *Eruca*, n = 1; *Lactuca*, n = 3; *Solanum*, n = 3; and *Spinacia*, n = 3), *Brassica* and *Solanum* showed the highest average alkane (18.3 and 25.6 μg/cm^2^, respectively) and total wax concentrations (36.5 and 37.3 μg/cm^2^, respectively; S2 Table). Nonacosane (C29), which was prevalent in *Brassica* vegetables, and hentriacontane (C31), which was the dominant alkane in *Solanum* crops (tomatoes), were different among the studied cultivars. The *Chchorium* genus showed the lowest average alkane and total wax concentrations (1.0 and 5.2 μg/cm^2^, respectively). The *Lactuca* genus showed the highest alcohol concentrations (8.6 μg/cm^2^), which accounted for 72% of the total wax. The primary form of wax in the *Lactuca* genus was the C26 alcohol, hexacosanol. *Brassica* vegetables including collards, kales, and cabbages, in addition to alkanes, fatty acids and alcohols, have high amounts of ketones (18.3 μg/cm^2^), which accounted for 33% of total epicuticular wax content among cultivars in this genus. The major ketone found in most *Brassica* vegetables was identified as nonacosan-15-one by the characteristic ketone fragments *m/z* 225 [CH_3_(CH_2_)_13_CO]^+^ and *m/z* 241 [36].

The *Brassica* genus cultivars commonly contain C29 alkane (nonacosane) and C29 ketone (nonacosanone) as the major epicuticular wax components (S1 Fig) Tomato (*Solanum lycopersicum*) fruits have the C31 alkane (hentriacontane) as the major compound and also comparable concentrations of the C29 (nonacosane) and C34 (tetratriacontane) alkane waxes. Lettuce (*Lactuca sativa*) leaf surfaces are dominated by C26 alcohol (hexacosanol) wax. In spinach (*Spinacia spp*.) the C31 alkane (hentriacontane) was the major wax compound. Leaves from the *Chchorium* genus cultivars contained C28 (octacosanol) and C30 (triacontanol) alcohols. Arugula (*Eruca* genus) contains C24 (tetracosanoic acid) fatty acid as the major wax compound. Epicuticular wax composition can be utilized as a method to assign plant species to specific taxon (chemotaxonomy) [46].

### SEM images of epicuticular surfaces

**Fig 2:**
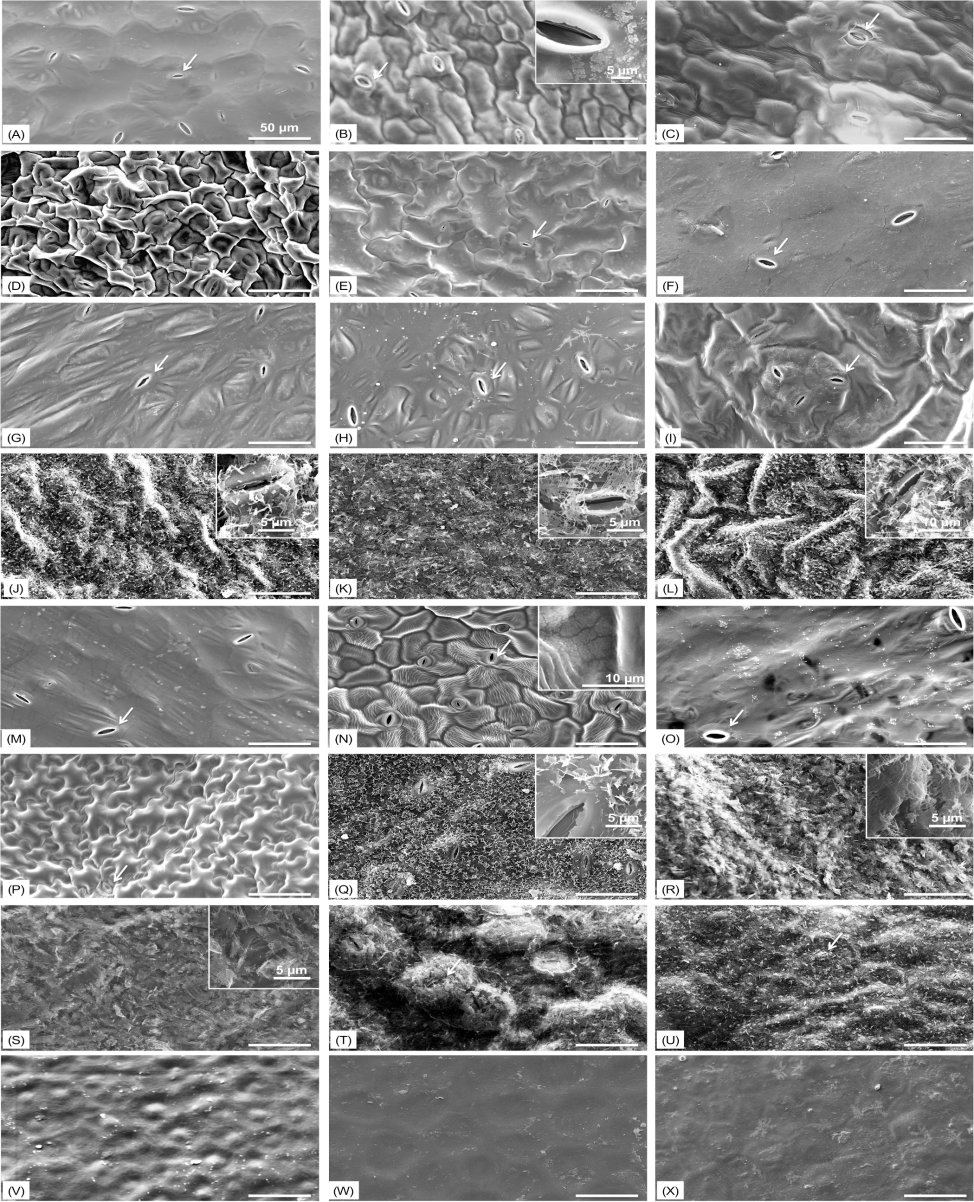
Epicuticular images from various vegetable leaves and tomato fruits. All SEM images were generated at 500 × resolution. Scale bar in the image is 50 μm. Inset images were taken at higher resolutions. White arrows indicate stomata. Alphabetical order matches sample list from Table 1. A: Tokyo bekana; B: ‘Perseo’ radicchio; C: ‘Rhodos’ endive; D: ‘Southern Giant Curled’ mustard; E: Mizuna; F: ‘Tyee’ spinach; G: ‘Racoon’ spinach; H: ‘Carmel’ spinach; I: Tatsoi; J: ‘Top Bunch’ collard; K: ‘Starbor’ kale; L: ‘Red Russian’ kale; M: Arugula; N: ‘Totem’ Belgian Endive; O: ‘Two Star’ lettuce; P: ‘Tropicana’ lettuce; Q: ‘Outredgeous’ romaine lettuce; R: ‘Super Red’ cabbage; S: ‘Gonzales’ cabbage; T: ‘Ruby Perfection’ cabbage; U: ‘Alcosa’ cabbage; V: ‘Sun Gold’ cherry tomato; W: ‘Indigo Rose’ tomato; X: ‘Rose’ tomato.

From the surface images taken by SEM, we can clearly see 3-dimensional crystalline wax structures on the epicuticular layers of eight of the 24 cultivars (Fig 2). Density and morphology of these 3-D wax structures varied among these eight cultivars [Fig 2J–2L and 2Q–2U]. Surfaces of the other cultivars are coated with a two-dimensional wax film, which has been reported as one of the most common epicuticular wax morphologies [45]. Previous work has reported that ketone-containing epicuticular waxes can occur in different morphological forms such as transversally ridged, coiled, branched, or triangular rodlets, usually associated with a background epicuticular wax film, covering the cuticle surface below and between the 3-D wax structures [47]. Collards, kale, and cabbage leaves have high ketone levels (nonacosan-15-one; Table 2) and these ketone waxes are probably responsible for the distinct crystal shapes. The ‘Outredgeous’ romaine lettuce had platelet-type 3-D wax crystals [Fig 2Q]. ‘Two Star’ and ‘Tropicana’ lettuce showed 2-D wax films on their surfaces at the vegetative stage, while leaves at the flowering stage possessed 3-D wax crystals.

**Fig 3:**
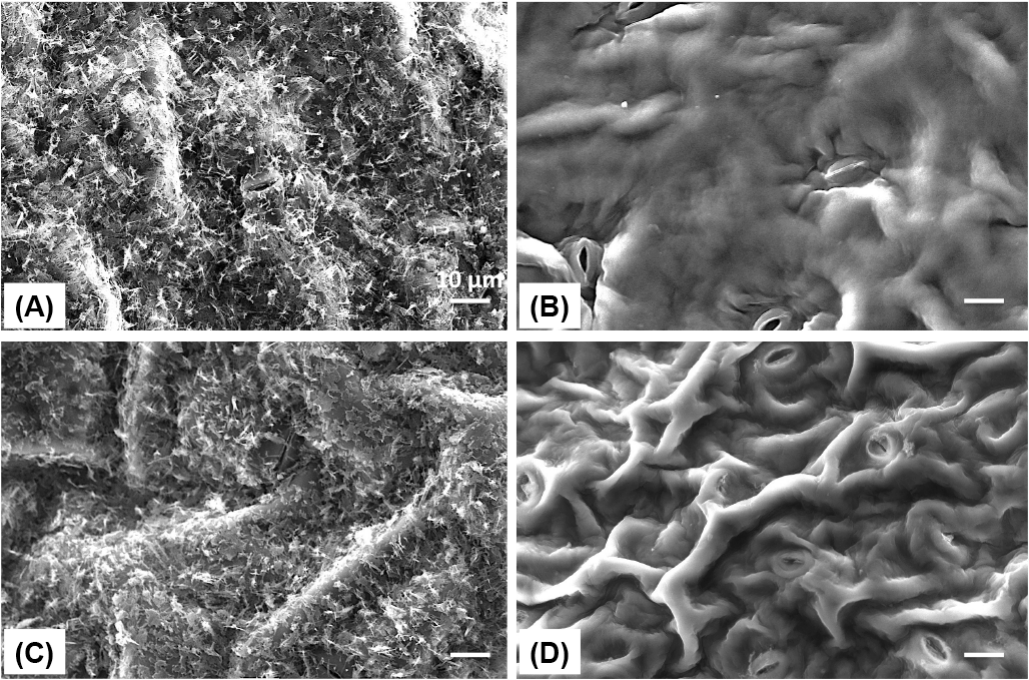
SEM images of epicuticular surfaces before and after 1 min chloroform extraction for ‘Top Bunch’ collards and ‘Starbor’ kale. A and B are intact ‘Top Bunch’ leafy collards before and after 1 min chloroform extraction, respectively. C and D are intact ‘Starbor’ leafy kale before and after 1 min chloroform extraction, respectively. Scale bar in the image is 10 μm.

We also observed that endive displayed the fewest stomata over 0.09 mm^2^ while collards had the most (Table 1). While tomato fruits do not have stomata, other cultivars show open stomata (Fig 2). Some leafy cultivars, including cabbage, kale, and collards showed wax crystals near or on stomata. The average lengths of stomata ranged from 7.5 to 22.3 μm. ‘Starbor’ kale had the smallest stomata while arugula had the largest (Table 1). Optical microscopy was used to determine the number of stomata. In a 0.09 mm^2^ of leaf surface area from different vegetables, the number of stomata ranged from 1 to 30. “Top Bunch” collards and ‘Starbor’ kale cultivars were used to generate epicuticular wax images before and after chloroform extraction to confirm epicuticular wax extraction method (Fig 3). One minute of chloroform extraction effectively removed all epicuticular waxes from the leaf surface. After chloroform extraction, stomata and cuticular roughness of leaf surfaces were distinctively visualized.

### OSU rotavirus adsorption to epicuticular surfaces

As confirmed by RT-qPCR results, OSU rotaviruses were found on the adaxial surfaces of all 24 cultivars when the produce surfaces were incubated with the viral suspension for 2 h at room temperature. The number of adsorbed rotaviruses on the leaf or tomato fruit surfaces (an area with a diameter of 11 mm) ranged from approximately 3.7 to 5.6 log_10_ genome copies/cm^2^ (Table 2). The three species with the highest levels of viral adsorption included ‘Southern Giant Curled’ mustard greens (5.6 ± 0.1 log_10_ genome copies/cm^2^), Tatsoi (5.4 ± 0.1 log_10_ genome copies/cm^2^), and ‘Racoon’ spinach (5.6 ± 0.2 log_10_ genome copies/cm^2^). The three species with the lowest the number of rotaviruses adsorbed on epicuticular surfaces were ‘Top Bunch’ collard (3.7 ± 0.1 log_10_ genome copies/cm^2^), ‘Sun Gold’ tomato (3.9 ± 0.4 log_10_ genome copies/cm^2^) and ‘Outredgeous’ romaine lettuce (4.1 ± 0.5 log_10_ genome copies/cm^2^). Within the *Solanum* genus, the cultivar ‘Rose’ had the highest number of adsorbed rotaviruses (4.4 ± 0.3 log_10_ genome copies/cm^2^), followed by ‘Indigo Rose’ (4.2 ± 0.4 log_10_ genome copies/cm^2^), and ‘Sun Gold’ tomatoes (3.9 ± 0.4 log_10_ genome copies/cm^2^). The percentage of rotaviruses that adhered to each cultivar was calculated using the numbers of rotaviruses adsorbed on the produce surfaces divided by the rotavirus genome copies in the initial virus suspension. From 0.1% to 10.8% of the initial viruses were found on produce surfaces, suggesting that the surface physicochemical properties of the produce may play an important role on viral adsorption. Control experiments with ‘Stabor’ kale and ‘Red Russian’ kale showed that viral adsorption was statistically similar for adaxial and abaxial surfaces (P = 0.89 for ‘Stabor’ kale; P = 0.18 for ‘Red Russian’ kale).

### Correlations within and between physicochemical properties of epicuticle and viral adsorption

Based on the statistical analysis, 16 significant correlations were found among and between physicochemical properties of epicuticular layer and the number of adsorbed rotaviruses (Table 3). Correlations within physicochemical properties of the produce were conducted from data generated from produce collected at the same time. Contact angle showed significant positive correlations with alkane (r = 0.659, P < 0.001), fatty acid (r = 0.442, P = 0.031), ketone (r = 0.637, P < 0.001), and total wax concentrations (r = 0.647, P < 0.001). Different wax concentrations were positively and significantly correlated with each other. Previous research that used leeks as a model found that epicuticular wax biosynthesis is initiated by the conversion of fatty acids to aldehydes, then alkanes, alcohols, and ketones [48]. This shared biosynthetic pathway would explain the co-correlation of the concentrations of various waxes.

**Table 3:** Correlation coefficients (r) between epicuticular physiochemical properties and the number of rotaviruses adsorbed on the produce surfaces.

	1	2	3	4	5	6	7	8	9
1. Contact angle (°)	1.000								
2. Surface roughness (μm)	-0.169								
3. No. of stoma	0.393	0.174							
4. Stoma length (μm)	-0.225	**0.516**	0.101						
5. Alkanes (μg/cm^2^)	**0.659**	-0.247	0.370	-0.065					
6. Fatty acids (μg/cm^2^)	**0.442**	0.221	**0.637**	**0.535**	**0.701**				
7. Alcohols (μg/cm^2^)	-0.418	-0.202	-0.292	-0.019	-0.179	-0.237			
8. Ketones (μg/cm^2^)	**0.637**	**-0.420**	0.074	-0.398	**0.809**	0.317	-0.071		
9. Total wax (μg/cm^2^)	**0.647**	-0.303	0.300	-0.123	**0.974**	**0.642**	-0.055	**0.899**	
10. Log_10_ viral adsorption (genome copies/mL)	-0.019	0.360	-0.356	0.112	**-0.498**	**-0.466**	-0.226	-0.246	**-0.473**

Contact angle, surface roughness, No. of stomata, stoma length, and wax extraction from the produce surface. N = 24 except for the correlations involving numbers and lengths of stomata (N = 21). Pearson’s correlation coefficients (r) were calculated by mean values of each variables from each cultivar, and the r values in bold are significantly correlated at P < 0.05.

**Fig 4:**
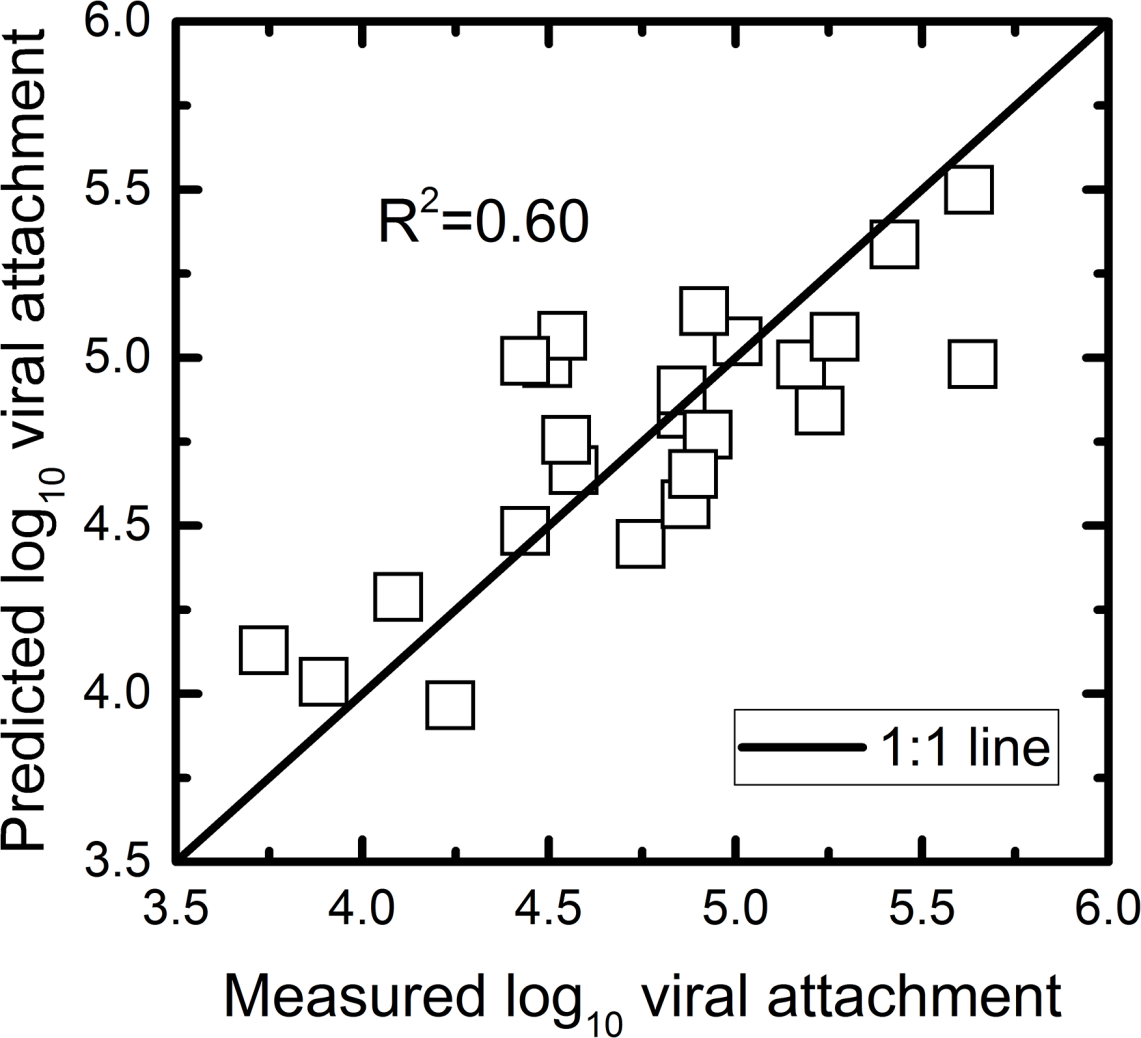
Partial least squares prediction model for the number of adsorbed viral particles on produce surfaces using six epicuticular physicochemical properties, including concentrations of alkanes, fatty acids, alcohols, and ketones, contact angle, and surface roughness.

Three significant correlations were found between the number of adsorbed rotaviruses and physicochemical properties of the epicuticle. The numbers of OSU rotaviruses adsorbed on the produce surfaces showed significant negative correlations with alkane (r = -0.498, P = 0.013), fatty acid (r = -0.466, P = 0.022), and total wax concentrations (r = -0.473, P = 0.020). Contact angle (r = -0.019, P = 0.930), surface roughness (r = 0.360, P = 0.084), stoma numbers (r = -0.356, P = 0.089), stoma lengths (r = 0.112, P = 0.518), alcohols (r = -0.226, P = 0.195), and ketones (r = -0.246, P = 0.174) were not correlated with the number of adsorbed rotaviruses. The six major epicuticular variables, alkane, fatty acid, alcohol, and ketone concentrations, contact angle, and surface roughness, were used to generate a PLS model (Fig 4) to predict the number of rotaviruses adsorbed on the epicuticular surfaces. Total wax was excluded because this is a redundant indicator for individual wax components. Stomata lengths and numbers were also excluded because viral adsorption was found on stomata-free tomato fruits. The performance of the final PLS model is evaluated according to the coefficient of determination (R^2^) and the root mean square error of prediction (RMSEP) in the prediction set. Generally, R^2^, which describes how well the data of the training set is mathematically reproduced, varies between 0 and 1 (with 1 indicating a perfectly fitted model). In PLS model six factors were extracted to get a maximized prediction value as the van der voet T^2^ statistic tests did not differ significantly from the optimized model (3 factors extraction, R^2^ = 0.60) with the minimum predicted residual sum of squares (PRESS) value. As RMSEP is a good measure of how accurately the model predicts the response, lower values of RMSEP indicate a better fit. A VIP score indicates how important this factor contributes to describing the variation in viral adsorption to vegetable surfaces, compared to other variables. A VIP ≥ 0.8 is considered the cut-off value for a variable making a significant contribution to dimensionality reduction [44]. We found that the RMSEP was 0.25 when 6 PLS factors were extracted in the prediction model and the PLS model explained 60% (adjusted R^2^ = 0.60) of the experiment-wide variation in the number of adsorbed rotaviruses using the physicochemical data. The alkane concentration showed the highest variable importance for projection value (VIP = 1.15), followed by fatty acids (1.12), contact angle (0.97), ketones (0.95), alcohols (0.92), and surface roughness (0.85).

## Discussion

In this study, the influence of 3-D epicuticular wax crystals, the chemical components of epicuticular layers, hydrophobicity and roughness of the produce surfaces, and the presence of stomata were investigated to reveal major surface properties associated with the number of adsorbed rotaviruses. Significantly, negative correlations between viral adsorption and the concentrations of alkanes, fatty acids, and total wax on the epicuticular surface were observed (Table 3). Although concentrations of alkanes, fatty acids, and total wax were significantly correlated with contact angle, which is a measurement of surface hydrophobicity, this trait was not correlated with the number of adsorbed rotaviruses. The lack of correlation with contact angle implies that the inhibition effects of the epicuticular wax components on viral adsorption may not be directly associated with increased hydrophobicity of the surfaces, but rather by the presence of 3-D epicuticular wax crystals. Indeed, the presence of 3-D wax crystals on the epicuticular layers of the produce showed significantly lower rotavirus adsorption than those without 3-D crystalized wax structures (P = 0.012; Table 4). For example, we observed a significantly lower number of rotaviruses adsorbed on the epicuticular surfaces on ‘Outredgeous’ romaine lettuce than on the other two lettuces (‘Two star’ and ‘Tropicana’). While these three lettuce cultivars had similar adaxial contact angles, only ‘Outredgeous’ romaine lettuce had 3-D epicuticular wax crystals. These results suggest that the presence of the 3-D epicuticular wax crystals may play a more important role in viral adsorption than surface hydrophobicity. Another explanation of the lack of correlation between OSU viral adsorption and hydrophobicity is that the measurement of contact angle was likely influenced by both surface hydrophobicity and roughness [49, 50].

**Table 4:** Comparison of physiochemical epicuticular properties between cultivars with 2-D or 3-D wax crystals on leaf surfaces.

Variable	2-D wax	3-D wax	P-value
No. of cultivars	13	8	
Contact angle	77.3 ± 5.9	106.1 ± 8.5	**0.010**
Surface roughness	4.1 ± 0.5	2.0 ± 0.2	**0.003**
No. of stoma	10.3 ± 1.8	18.7 ± 2.5	0.425
Stoma length	13.6 ± 1.2	11.2 ± 1.1	0.180
Alkanes	2.7 ± 0.6	24.2 ± 6.6	**0.005**
Fatty acids	2.0 ± 0.4	5.0 ± 0.8	**0.003**
Alcohols	2.7 ± 0.4	3.6 ± 1.7	0.521
Ketones	0.10 ± 0.04	16.50 ± 4.80	**<0.001**
Total wax	7.6 ± 0.8	49.5 ± 11.4	**<0.001**
Log_10_ viral adsorption	6.43 ± 0.12	5.97 ± 0.14	**0.012**

Absence or presence of 3-D wax crystals was determined by SEM. LSD test was used to indicate significant difference for the variables between cultivars with and without wax crystals on leaf surface. Tomato cultivars were excluded because of different tissue type.

Although previous work suggested that surface roughness favors microorganism adhesion and prevents detachment of *E*. *coli* from selected fruits and sprouting seeds when treated with a combination of organic acids and surfactants [51, 52], the smaller size of OSU rotaviruses compared to bacteria may allow for viral adsorption on the produce surfaces regardless of the roughness. Using the dynamic light scattering method described in previous studies [53, 54], we found that the rotavirus suspension showed one peak with an average diameter of 175 ± 1 nm, suggesting of a slight aggregation of every two viruses. This aggregation size is much lower than the height variations of adaxial surfaces (1.1 μm to 8.0 μm) within the 24 cultivars from various species. We suggest that the relatively smaller size of rotaviruses compared to the height variations of the leaf surfaces allow the adsorbed rotaviruses to be located in the “valleys” of produce surfaces and may not be removed by the washing treatments. Our results suggest that for nanometer-sized viruses, compared to micrometer scale bacteria like *E*. *coli*, surface roughness may not be a critical factor controlling viral adsorption to produce surfaces.

Previous work found the aggregation of *E*. *coli* O157 and norovirus virus-like-particles on or inside the stomata [13, 19], suggesting that the presence of stomata may significantly help viruses adsorb to the vegetable surfaces. Hence, the contribution of stomata to viral adsorption was also investigated in our study by correlating adaxial stoma numbers and lengths with the number of adsorbed rotaviruses. While no significant correlations were found between the numbers of adsorbed rotaviruses and adaxial stoma numbers (P = 0.113) and lengths (P = 0.689), we found that vegetable samples with crystalized wax present on their surfaces showed significantly higher contact angles, and concentrations of alkanes, fatty acids, ketones, and total wax, as well as significantly lower surface roughness and the number of adsorbed rotaviruses, than the samples without 3-D crystalized wax present on the epicuticular surface (Table 4). This observation is consistent with a previous study reporting a reduced adsorption of the plant fungal pathogen, *Agathis robusta*, when stomata were covered by wax [55]. In our study, eight of the 24 vegetables had 3-D wax crystals on their epicuticular layers, and seven out of eight had stomata covered by wax crystals, as shown in Fig 2. The wax crystals shielding the stomata could prevent OSU rotaviruses from residing on or inside the stomata. Notably, ‘Outredgeous’ romaine lettuce did not have stomata covered by wax crystals, suggesting the potential for deposition of rotaviruses on or inside the stomata as observed in a previous study showing norovirus-like-particle aggregation at stomata of romaine lettuce leaves [19]. In addition, up to 4.4 ± 0.3 log_10_ genome copies/ cm^2^ OSU rotaviruses were able to adsorb to tomato fruit surfaces, which do not have stomata [56]. These results suggest that for this comprehensive set of 24 fresh produce cultivars the presence of stomata is not necessary for rotavirus adsorption to produce surfaces, and the presence of 3-D epicuticular wax crystals covering stomata, rather than the stoma lengths and numbers, may play a more important role in the number of adsorbed rotaviruses associated with leaf stomata.

Electrostatic forces, the presence of stomata, and exposed carbohydrates on epicuticular surfaces of plants have been suggested as important contributors to the number of rotaviruses adsorbed on the produce surfaces [15, 18, 19]. Here we established a PLS prediction model to quantitatively explain the number of rotaviruses adsorbed on the epicuticular surfaces based on the physicochemical properties of the epicuticular surfaces. The PLS model was selected instead of multiple linear regression model because PLS allows for the inclusion of X variables that co-correlate [57]. As described above, significant correlations between contact angles and concentrations of alkane, ketones, and fatty acids were observed. Based on the PLS model results, the major epicuticular properties which included the concentration of alkanes, fatty acids, alcohols, and ketones, contact angle, and surface roughness, together could explain 60% of the variation in viral adsorption among the cultivars. While we found moderate correlations between each individual variable and the number of adsorbed rotaviruses, none of these factors can individually explain more than 25% of the viral adsorption results. The highest coefficient of determination was observed between viral adsorption and alkane concentrations (R^2^ = 0.238). The PLS model results suggest that these major epicuticular properties together impact the number of adsorbed rotaviruses. In addition, these major epicuticular properties are interdependent. For example, increasing wax contents may generate physical barriers that can increase contact angle. To the best of our knowledge, statistical modeling for prediction of viral adsorption has not been conducted, and this study for the first time calculated how these produce surface variables could quantitatively describe viral adsorption.

In summary, OSU rotaviruses were found to attach to a wide range of salad vegetables, suggesting a potential public health concern regarding rotavirus contamination during fresh produce pre-harvest production. This is the first report of lower viral adsorption on fresh produce surfaces associated with the presence of 3-D epicuticular wax crystals. In addition, the results obtained with 24 cultivars of leafy vegetables and tomato fruits commonly used in salads suggest that physical and chemical surface properties of the fresh produce need to be collectively considered for efficient sanitizer development. Future studies will determine whether commonly used sanitation strategies effectively remove adsorbed viruses and how these strategies influence the concentrations of alkanes, fatty acids, alcohols, and ketones on the produce surfaces that may allow for recontamination after sanitation.

## Supporting Information

S1 Fig GC-FID chromatograph for the wax analysis. (PDF)

S1 Table Sample list and botanical information. (Contact angle is presented in °, and roughness is in μm.). Ad and ab indicate adaxial and abaxial leaf, respectively. Stoma lengths were measured on adaxial leaf surfaces.
(PDF)

S2 Table Epicuticular properties of each plant genus. Mean ± SEM.
(PDF)
