# Supplementary figures and images for: Influence of Epicuticular Physicochemical Properties on Porcine Rotavirus Adsorption to 24 Leafy Green Vegetables and Tomatoes
Source: PLoS One. 2015 Jul 16;10(7):e0132841. doi: 10.1371/journal.pone.0132841 (PMC4504507; doi:10.1371/journal.pone.0132841)

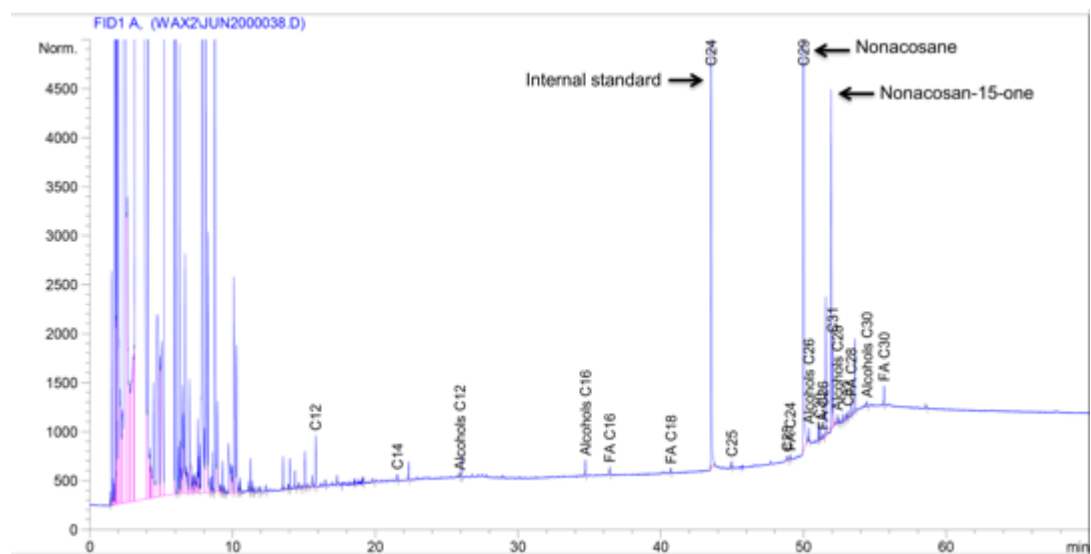

Figure 1S. GC-FID chromatograph for the wax analysis.

Supplement: S1 Fig — (PDF) [file pone.0132841.s001.pdf]
