# Supplementary material for: Influence of Epicuticular Physicochemical Properties on Porcine Rotavirus Adsorption to 24 Leafy Green Vegetables and Tomatoes
Source: PLoS One. 2015 Jul 16;10(7):e0132841. doi: 10.1371/journal.pone.0132841 (PMC4504507; doi:10.1371/journal.pone.0132841)
Supplement: S1 Table — (Contact angle is presented in °, and roughness is in μm.). Ad and ab indicate adaxial and abaxial leaf, respectively. Stoma lengths were measured on adaxial leaf surfaces. (PDF) [file pone.0132841.s002.pdf]

Table S1. Sample list and botanical information.

Johnney's Selected Seeds catalog no.	Genus	Species	Species/cultivar	Tissue used	Maturity (days)
2251.25	*Brassica*	*rapa*	Tokyo bekana	Leaf	45
3099.52	*Cichorium*	*intybus*	'Perseo' radicchio	Head	55
339.53	*Cichorium*	*endivia*	'Rhodos' endive	Leaf	42
377X.26	*Brassica*	*juncea*	'Southern Giant Curled' mustard	Leaf	45
2883.26	*Brassica*	*rapa*	Mizuna	Leaf	40
646.54	*Spinacia*	*oleracea*	'Tyee' spinach	Leaf	40
2550.54	*Spinacia*	*oleracea*	'Racoon' spinach	Leaf	34
665.11	*Spinacia*	*oleracea*	'Carmel' spinach	Leaf	35
2897.11	*Brassica*	*rapa*	Tatsoi	Leaf	45
2190.25	*Brassica*	*oleracea*	'Top Bunch' collards	Leaf	50
305.53	*Brassica*	*oleracea*	'Starbor' kale	Leaf	55
363.26	*Brassica*	*napus*	'Red Russian' kale	Leaf	50
385.26	*Eruca*	*sativa*	Arugula	Leaf	40
2320G.52	*Cichorium*	*intybus*	'Totem' Belgian Endive	Leaf	50
451GP.11	*Lactuca*	*sativa*	'Two Star' lettuce	Leaf	50
2485.11	*Lactuca*	*sativa*	'Tropicana' lettuce	Leaf	52
2208N.26	*Lactuca*	*sativa*	'Outredgeous' romaine lettuce	Leaf	57
168.53	*Brassica*	*oleracea*	'Super Red' cabbage	Head	78
2907.53	*Brassica*	*oleracea*	'Gonzales' cabbage	Head	66
177.53	*Brassica*	*oleracea*	'Ruby Perfection' cabbage	Head	85
2985.11	*Brassica*	*oleracea*	'Alcosa' cabbage	Head	72
770.5	*Solanum*	*lycopersicum*	'Sun Gold' cherry tomatoes	Fruit	57
3616.51	*Solanum*	*lycopersicum*	'Indigo Rose' tomatoes	Fruit	75
2166.51	*Solanum*	*lycopersicum*	'Rose' tomatoes	Fruit	78
