# Supplementary material for: Influence of Epicuticular Physicochemical Properties on Porcine Rotavirus Adsorption to 24 Leafy Green Vegetables and Tomatoes
Source: PLoS One. 2015 Jul 16;10(7):e0132841. doi: 10.1371/journal.pone.0132841 (PMC4504507; doi:10.1371/journal.pone.0132841)
Supplement: S2 Table — Mean ± SEM. (PDF) [file pone.0132841.s003.pdf]

Table S2. Epicuticular property of each plant genus.

Genus (no.)	*Brassica* (11)	*Cichorium* (3)	*Eruca* (1)	*Lactuca* (3)	*Solanum* (3)	*Spinacia* (3)
Contact angle	104.1 ± 5.6	54.1 ± 1.2	92.4	54.3 ± 3.2	98.0 ± 7.3	97.1 ± 4.9
Surface roughness	3.0 ± 0.7	4.5 ± 0.5	5.3	2.7 ± 0.1	4.7 ± 1.8	3.1 ± 0.2
No. of stoma	18.3 ± 2.4	5.0 ± 2.7	9.3	8.7 ± 3.5	-	10.6 ± 1.7
Stoma length	10.9 ± 0.9	14.7 ± 1.8	22.3	16.0 ± 1.7	-	10.8 ± 0.3
Alkanes	18.3 ± 5.6	1.0 ± 0.3	2.2	1.1 ± 0.3	25.6 ± 6.0	6.3 ± 0.3
Fatty acids	4.2 ± 0.8	1.5 ± 0.3	6.4	2.1 ± 0.7	10.3 ± 4.1	1.1 ± 0.1
Alcohols	1.8 ± 0.3	2.8 ± 0.3	1.3	8.6 ± 3.4	0.9 ± 0.5	2.9 ± 0.1
Ketones	12.0 ± 4.1	0.0 ± 0.0	0.5	0.1 ± 0.1	0.4 ± 0.2	0.0 ± 0.0
Total wax	36.5 ± 10.5	5.2 ± 0.9	10.4	12.0 ± 4.3	37.3 ± 9.0	10.4 ± 0.6
Log_{10} viral attachment	6.2 ± 0.2	6.3 ± 0.2	6.4	5.9 ± 0.2	5.6 ± 0.2	6.6 ± 0.2

Mean ± SE
